# Real-Time Monitoring of Colorectal Cancer Location and Lymph Node Metastasis and Photodynamic Therapy Using Fucoidan-Based Therapeutic Nanogel and Near-Infrared Fluorescence Diagnostic–Therapy System

**DOI:** 10.3390/pharmaceutics15030930

**Published:** 2023-03-13

**Authors:** Yoo-kyoung Shin, You-rim Park, Hyeri Lee, Yongdoo Choi, Joo Beom Eom

**Affiliations:** 1 Department of Biomedical Science, College of Medicine, Dankook University, Cheonan 31116, Republic of Korea; 2 Division of Technology Convergence, Research Institute, National Cancer Center, 323 Ilsan-ro, Goyang 10408, Republic of Korea

**Keywords:** photodynamic therapy, real-time monitoring, image-guided surgery, lymph node metastasis, colorectal cancer, fluorescence imaging

## Abstract

We report real-time monitoring of colorectal cancer, lymph node metastasis of colorectal cancer cells, and tumor growth inhibition through photodynamic therapy (PDT) using a near-infrared fluorescence diagnostic–therapy system with a light source for PDT and a fucoidan-based theranostic nanogel (CFN-gel) with good accumulation efficiency in cancer cells. To confirm the effect of the fabricated system and developed CFN-gel, in vitro and in vivo experiments were performed. Chlorin e6 (Ce6) and 5-aminolevulinic acid (5-ALA) were used for comparison. We confirmed that CFN-gel has a high accumulation efficiency in cancer cells and high fluorescence signals in near-infrared light for a long period, and only CFN-gel delayed the growth rate of cancer in terms of its size in PDT. In addition, using the near-infrared fluorescence diagnostic–therapy system and CFN-gel prepared for these experiments, the lymph node metastasis of cancer cells was imaged in real time, and the metastasis was confirmed through H&E staining. The possibility of image-guided surgery and identification of lymph node metastasis in colorectal cancer can be confirmed through CFN-gel and a near-infrared fluorescence diagnostic–therapy system that includes various light sources.

## 1. Introduction

It was estimated that one in six deaths was caused by cancer, and the disease was responsible for nearly 10 million deaths in 2020 [1]. The most common types of cancer are breast, lung, colon, rectal, and prostate cancers, which slightly vary between countries [1,2]. The cancer incidence rate is rapidly growing worldwide, and cancer is an important barrier to increasing life expectancy [2,3]. Many methods have been studied in recent decades to overcome this deadly disease. As a result, cancer treatments such as surgery, radiation therapy, chemotherapy, molecular targeted therapy, and immunotherapy have made significant progress and still hold potential. However, these methods still have limitations including severe side effects, invasiveness, and the inability to effectively treat late stages [4,5]. In the case of chemotherapy, side effects on the skin and hair, bone marrow and blood, gastrointestinal tract, and kidneys have been reported [6]. In radiation therapy, skin erythema, mucositis, nausea, diarrhea, radiation-induced fibrosis, atrophy, vascular damage, and neural damage have been reported [7]. Therefore, a new cancer treatment method to overcome the side effects is needed. PDT is a promising new strategy for cancer treatment, and many research groups are currently conducting research on this treatment [8]. Photosensitizers (PSs) become a triplet state that can have electron activity for a relatively long time in the absence of acceptors, and the activated triplet causes two kinds of reactions. Excited PS triplet-state electrons produce reactive oxygen species (ROS) or singlet-state oxygen species (^1^O_2_) depending on the surrounding oxygen concentration [9]. Both mechanisms of action generally co-occur and induce apoptosis or necrosis by oxidative damage to surrounding proteins, lipids, and nucleic acids [8,10,11,12,13]. PDT has several advantages, such as minimal invasiveness, localized treatment, and possible time-selective treatment, which generates ROS only when light irradiates.

Furthermore, PDT provides better selectivity for the targeting of cancers than conventional cancer treatment because of the preferential accumulation of PSs in cancers [14,15]. Another advantage of PDT is that optical imaging is possible using the fluorescence properties of PSs. Optical imaging using the fluorescence properties of PSs plays an important role in cancer diagnosis and treatment. PSs tend to accumulate in neoplastic tissues and emit light upon activation. Accurately defining tumor boundaries between cancerous and normal tissues is critical for photodynamic diagnosis (PDD) and fluorescence-guided resection (FGR) treatment because excessive resection of normal tissue can seriously affect patients. Optical fluorescence imaging using PSs quantitatively shows changes in fluorescence before and after treatment and is crucial in monitoring treatment effects [16,17,18]. The simultaneous progress of fluorescence imaging and PDT using these characteristics of PSs has many advantages over conventional diagnosis and treatment methods [4]. Existing diagnostic methods, such as white light endoscopy, lack overall sensitivity, limit their detection ability, and limit their use for therapeutic purposes [19]. A system that can simultaneously perform treatment and diagnosis is required to achieve early diagnosis and treatment of cancer. In addition, a new system capable of image acquisition and treatment of PSs of various wavelengths is required.

In this study, we report a near-infrared fluorescence diagnostic–therapy system designed for tumor monitoring and effective treatment. The optical paths of the fluorescence absorption and treatment light sources are not only configured to match using a diode and multimode fiber-based switch but also designed to be easily replaced so that they can be applied to various PSs. Using this system, diagnosis and treatment were simultaneously performed in the same optical path using 5-ALA [20], Ce6 [21,22], and CFN-gel [23]. Colorectal cancer cells [24,25,26] were selected to confirm the performance of the proposed system and CFN-gel developed in previous studies with a longer blood circulation time than conventional PSs. This is because it takes a lot of time from the indication of colorectal cancer to surgery, so diagnosis/treatment is possible only when the fluorescence accumulated during that time remains [27,28]. In addition to the proposed CFN-gel, 5-ALA and Ce6, which are commercially available, were used and compared. Confocal and two-photon microscopies were used to obtain the fluorescence images of colorectal cancer cells treated with 5-ALA, Ce6, and CFN-gel. The accumulation efficiencies and fluorescence signals over time were compared. In addition, 5-ALA, Ce6, and CFN-gel used in the experiments were injected into a xenograft mouse model inoculated with colorectal cancer cells, and the location of the cancer and the lymph node metastasis of cancer cells were confirmed in real time using the proposed imaging system. Metastasis of cancer cells to the lymph nodes was verified by H&E staining. Finally, PDT was performed on the cancer model mice in which PpIX (5-ALA), Ce6, and CFN-gel accumulated one day later to confirm whether cancer growth was suppressed over time. Through experiments using the proposed system and CFN-gel, it was possible to confirm the possibility of precise treatment during colorectal cancer surgery in the future, which is expected to become a key technology for image-guided surgery.

## 2. Materials and Methods

### 2.1. Near-Infrared Fluorescence Diagnostic–Therapy System

**Figure 1:**
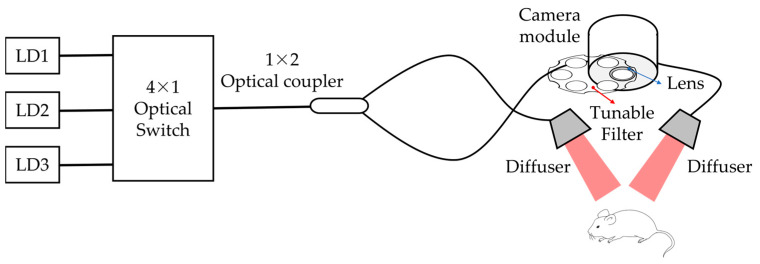
Schematic diagram of a developed near-infrared fluorescence diagnostic–therapy system.

Figure 1 shows a schematic of the developed near-infrared fluorescence diagnostic–therapy system. A multimode fiber-based switch (FOSW14N62L2, Lightwave Link Inc., Taiwan) was employed to integrate the light path used for various absorption and fluorescence light sources. In this study, three types of laser diodes, with wavelengths of 405 nm, 650 nm, and 660 nm, were used for activating PpIX (5-ALA), Ce6, and CFN-gel, respectively. The light sources from the optical switch were separated and irradiated through a 1 × 2 optical coupler, and the light source was dispersed using diffusers for uniform irradiation. The fluorescence camera module consisted of a lens and a bandpass filter. Bandpass filters with pass regions of 620 nm ± 30 and 670 ± 10 nm were used to block the light source and pass only the fluorescence wavelength, respectively. To provide a sufficient working distance, a lens with a focal length of 200 mm was used and mounted on the front of the camera module. Images acquired from the camera were transferred to a PC using a USB 3.0 port and stored.

### 2.2. Materials for PDT

The materials used in this experiment were 5-aminolevulinic acid (A0325, Tokyo Chemical Industry, Tokyo, Japan), Chlorin e6 (21684, Cayman Chemical, Ann Arbor, MI, USA), and CFN-gel (self-made, National Cancer Center, Gyeonggi-do, Republic of Korea). The preparation and characterization of CFN-gels were introduced in a previous study [23]. Briefly, the manufacturing method of CFN-gel is as follows. To prepare the Ce6–fucoidan conjugates, fucoidan (3.02 μmol) was dissolved in a sodium phosphate buffer (PBS; 10 mM, pH 7.4, 137 mM NaCl). Then, carbodiimide (EDC, 120 μmol) and sulfo-*N*-hydroxysuccinimide (sulfo-NHS, 125 μmol) were added to the fucoidan solution and reacted for 30 min. Then, PBS solution (1 mL) containing 120 μmol of cystamine dihydrochloride was added to the activated fucoidan solution. After 18 h, the solution was dialyzed against deionized (DI) water for purification and then freeze-dried. Next, Ce6 molecules were conjugated into the aminefunctionalized fucoidan. The carboxylic acid group of Ce6 (33.5 μmol) was activated using EDC (340 μmol) and sulfo-NHS (350 μmol) in a dimethyl sulfoxide solvent. Then, the activated Ce6 solution was added to amine-functionalized fucoidan in a DMF:H_2_O co-solvent (1:1 *v*/*v*). After 18 h, the purified Ce6–fucoidan conjugate (CFN-gel) was obtained after dialysis, and the final reactant solution was freeze-dried. The hydrodynamic size of CFN-gel is 259 nm [23].

### 2.3. Cell Line and Cell Culture

The HCT 116 human colorectal carcinoma cells were obtained from the Korean Cell Line Bank (KCLB, Seoul, Republic of Korea). These cell lines were cultured in RPMI1640, which contains 10% fetal bovine serum (FBS), penicillin (100 U/mL), and streptomycin (100 μg/mL). The cells were grown at 37 °C and 5% CO_2_ in a cell incubator.

### 2.4. Confocal/Two-Photon Fluorescence Microscope for Intracellular Uptake

HCT 116 cells (1 × 10^6^ cells/well) were seeded in FluoroDish (35 mm diameter (FD35-100, World Precision Inc., USA)) and incubated for 24 h for cell attachment. Then, the cells treated with 5-ALA (1 mM) [29,30], Ce6 (2 μM), and CFN-gel (2 μM Ce6 equivalent) [23] were incubated at 37 °C for 3, 6, 9, 12, and 24 h. After incubation, the cells were washed, and new culture media were added. NIR fluorescence images of PpIX (5-ALA) (λ_ex_ = 405 nm, λ_em_ = 635 nm), Ce6, and CFN-gel (λ_ex_ = 405 nm, λ_em_ = 670 nm) were acquired using a confocal microscope (FV-3000; Olympus, Japan) at 40× lens magnification. Two-photon microscopic images of the same cells were acquired using a two-photon microscope (homemade) with a tunable femtosecond laser (Chameleon Ultra II, Coherent, USA) with 750 nm excitation wavelength and 40× lens magnification. Quantitative analysis of the fluorescence intensity was conducted using MATLAB.

### 2.5. Cell Viability Assay

An experiment was conducted on HCT 116 colorectal cancer cells categorized into two groups to confirm the cancer cell treatment ability of PpIX (5-ALA), Ce6, and CFN-gel itself and the PDT effect in the cell state. In one group, only 5-ALA, Ce6, and CFN-gel were treated, and in the other group, PDT was performed after 5-ALA, Ce6, and CFN-gel treatment. First, HCT 116 cancer cells were seeded in 96-well plates at a density of 1 × 10^4^ cells/well and incubated for 24 h to allow cell attachment. The non-PDT group was treated with 5-ALA (0, 0.5, 1, 2, and 5 μM), Ce6 (0, 1, 2, and 5 μM), and CFN-gel (0, 1, 2, and 5 μM Ce6 equivalent) for 6 h. The PDT group was treated with 5-ALA (0, 0.5, 1, 2, and 5 μM), Ce6 (0, 1, 2, 3, and 5 μM), and CFN-gel (0, 1, 2, 3, and 5 μM Ce6 equivalent) for 6 h, after which a fresh cell culture medium was added. At this stage, the PDT group underwent PDT with a 660 nm CW laser at 9 J/cm^2^ (an irradiation dose rate of 50 mW/cm^2^). After incubating the cells for 24 h, cell viability was analyzed using the Cell Counting Kit-8 (CK04-11, Dojindo, Japan).

### 2.6. In Vivo NIR Fluorescence Imaging

In this experiment, 7-week-old male BALB/c nude mice were used. All experiments were conducted according to protocols approved by the Institutional Animal Care and Use Committee of Dankook University (DKU-21-072). For in vivo experiments, HCT 116 cells were subcutaneously transplanted into four BALB/c nude mice and grown until the tumors reached ~200 mm^3^ in size. To acquire real-time fluorescence images, each mouse was injected with PBS (100 μL), 5-ALA (250 mg/kg), Ce6 (5 mg/kg), and CFN-gel (5 mg Ce6 equivalent/kg), and fluorescence images were acquired using the proposed system. The light source for fluorescence imaging was 405 nm for mice injected with 5-ALA and 650 nm for mice injected with Ce6 and CFN-gel (an irradiation dose rate of 20 mW/cm^2^). Fluorescence images were acquired 4, 6, 9, and 24 hours after injecting 5-ALA, Ce6, and CFN-gel, respectively. After 24 h, the mice were sacrificed, and the cancers and organs were extracted to confirm the remaining PpIX (5-ALA), Ce6, and CFN-gel through fluorescence imaging. Lymph nodes were extracted to confirm the metastasis of colorectal cancer through fluorescence imaging using the proposed system and H&E staining.

### 2.7. In Vivo PDT

For PDT performance using the proposed system, BALB/c nude mice (Orientbio Inc., Republic of Korea) were divided into five groups: group 1 (control, no injection), group 2 (PBS), group 3 (5-ALA), group 4 (Ce6), and group 5 (CFN-gel). After injecting the HCT 116 cancer cell line into each group, tumors were grown to 100–200 mm^3^ in size. PBS (100 μL), 5-ALA (250 mg/kg), Ce6 (5 mg/kg), and CFN-gel (5 mg Ce6 equivalent/kg) were intravenously injected into the mouse models. After 24 h, the light source was changed to a 660 nm wavelength for PDT through the switch of the proposed system, and an energy of approximately 18 J/cm^2^ (irradiation for 6 min with an optical power of 50 mW/cm^2^) was applied to each of the tumors in groups 2–5. PDT was performed only once a day after the injection of 5-ALA, Ce6, and CFN-gel, and the tumor volume was observed for 12 days to compare the groups.

## 3. Results

### 3.1. Intracellular Accumulation of Photosensitizers

**Figure 2:**
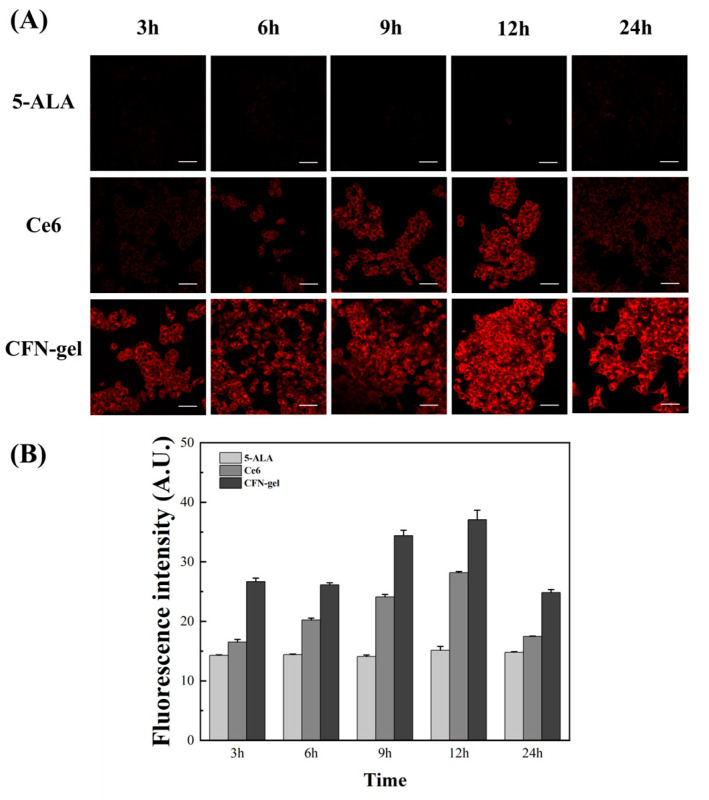
Time-lapse confocal microscopy images of 5-ALA-, Ce6-, and CFN-gel-treated HCT 116 cancer cells. (**A**) HCT 116 cancer cells treated with 5-ALA (1 mM), Ce6 (2 μM), and CFN-gel (2 μM Ce6 equivalent) for 3, 6, 9, 12, and 24 h. Images of 5-ALA-induced protoporphyrin IX (λ_ex_ = 405 nm, λ_em_ = 635 nm) and Ce6 and CFN-gel (λ_ex_ = 405 nm, λ_em_ = 670 nm) were obtained. (**B**) Quantitative analysis of fluorescence intensities in the 5-ALA-, Ce6-, and CFN-gel-treated HCT 116 cells. Scale bars represent 50 μm.

**Figure 3:**
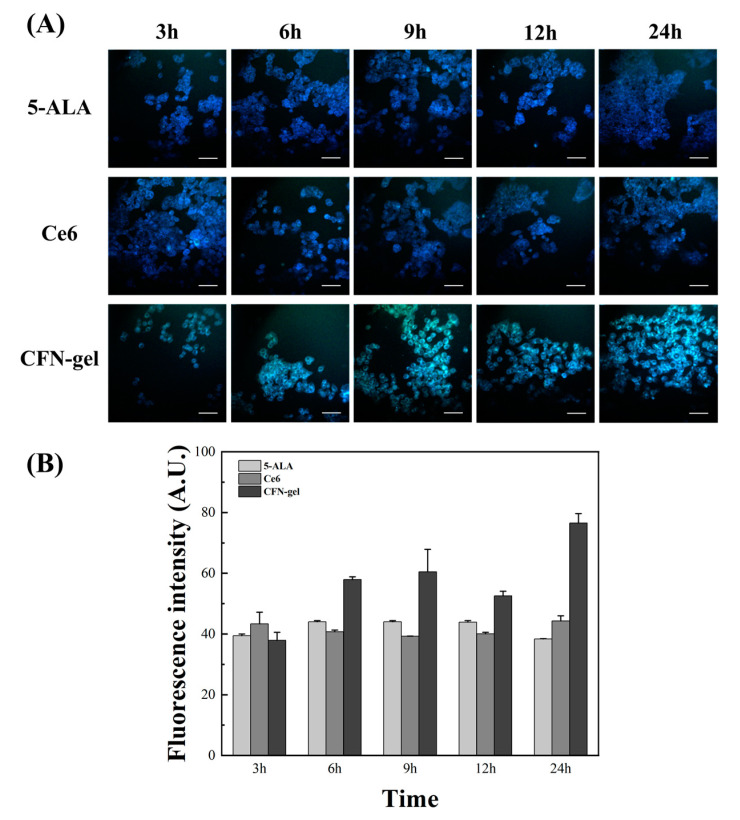
Time-lapse two-photon microscopy images of 5-ALA-, Ce6-, and CFN-gel-treated HCT 116 cancer cells. (**A**) HCT 116 cancer cells treated with 5-ALA (1 mM), Ce6 (2 μM), and CFN-gel (2 μM Ce6 equivalent) for 3, 6, 9, 12, and 24 h. Images were obtained at 750 nm excitation wavelength. (**B**) Quantitative analysis of fluorescence intensities in the 5-ALA-, Ce6-, and CFN-gel-treated HCT 116 cells. Scale bars represent 50 μm.

In order to more accurately confirm the accumulation of the contrast agent injected into the HCT 116 cancer cells and compare the images with a confocal microscope, images for 5-ALA, Ce6, and CFN-gel are shown in Figure 2 and Figure 3 using a self-made two-photon microscope. In the case of 5-ALA, the fluorescence intensity increased compared with the one-photon image, but it was found through the two-photon image that it was still lower than CFN-gel. However, although the fluorescence intensity was low, this contrast agent was also bound to cancer cells. Ce6 also seemed to initially bind well with cancer cells, but the fluorescence intensity was not very high over time. However, in the case of CFN-gel, the binding to colorectal cancer cells showed a low fluorescence intensity at first. Still, after 6 h, it remarkably increased, and even after 24 h, the fluorescence intensity was higher than that of 5-ALA and Ce6. As such, confirming the accumulation of PpIX (5-ALA), Ce6, and CFN-gel to cancer cells and the fluorescence intensity through one- and two-photon images was possible. In the case of CFN-gel, the fluorescence intensity is maintained even after 24 h. As such, the binding of PpIX (5-ALA), Ce6, and CFN-gel to cancer cells and the fluorescence intensity can be confirmed through one- and two-photon imaging. Because CFN-gel maintains fluorescence intensity even after 24 h, it is expected to be used to check the location of cancer in real time during colorectal cancer surgery.

### 3.2. In Vitro Cell Studies

**Figure 4:**
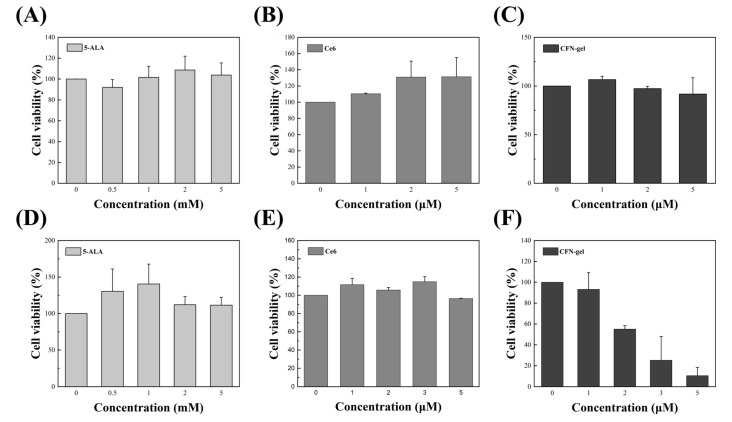
In vitro cell viability on HCT 116 cells treated with various 5-ALA, Ce6, and CFN-gel concentrations. Viability was measured by CCK-8 assay. (**A**–**C**) Concentration-dependent cancer cell treatment ability of PpIX (5-ALA), Ce6, and CFN-gel. (**D**–**F**) Cell viability of HCT 116 cells with 5-ALA-, Ce6-, and CFN-gel-mediated PDT using a laser diode (wavelength, 660 nm; fluence, 9 J/cm^2^).

To confirm the cancer cell treatment ability of PpIX (5-ALA), Ce6, and CFN-gel itself presented in this experiment, and the PDT effect in the cell state, 5-ALA, Ce6, and CFN-gel were prepared for each concentration. 5-ALA, Ce6, and CFN were treated with HCT 116 cancer cells at different concentrations. After 6 h, the cell viability of the group not irradiated with light and the group irradiated with light was confirmed. The light used for PDT had a wavelength of 660 nm and an irradiation dose of 9 J/cm^2^ (irradiation for 3 min with an optical power of 50 mW/cm^2^). Figure 4A–C shows the group that was not irradiated with light, and Figure 4D–F shows the group that performed PDT with light irradiation. In the group not irradiated with light, cancer cells rather increased, and CFN-gel showed a slight decrease, but the change was not so great. However, in each light-irradiated group, the number of cells slightly decreased or was maintained in the case of 5-ALA or Ce6, but in the case of the CFN-gel, it was found that the number of cancer cells rapidly decreased after PDT. The reason is that the amount of the cellular uptake of 5-ALA and CFN-gel is different, and the singlet oxygen quantum yield (0.6) and extinction coefficient (5000) of PpIX (5-ALA converts to PpIX inside cancer cells) are much smaller than those of Ce6 (0.8 and 40,000, respectively). As such, the possibility of PDT treatment with CFN-gel was confirmed even in the cellular state. In addition, as can be seen from the PDT results, it was confirmed that the survival rate of cancer cells significantly decreased when the concentration of CFN-gel was 2 μM or higher. The fluorescence imaging technology proposed through cell-level imaging and PDT experiments using a combination of cancer cells and 5-ALA, Ce6, and CFN-gel and the previously developed CFN-gel are expected to be the core of colorectal cancer image-guided surgery, as well as removing only the cancer cells. This possibility is confirmed through in vivo experiments in the next section.

### 3.3. In Vivo Fluorescence Imaging

**Figure 5:**
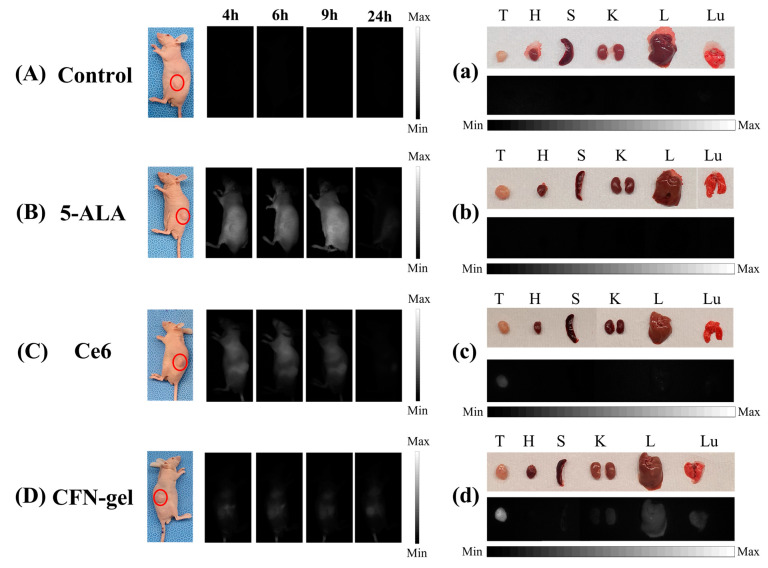
In vivo fluorescence imaging in xenograft tumor models. (**A**–**D**) Fluorescence images obtained after 4, 6, 9, and 24 h after intravenous injection of PBS (100 μL), 5-ALA (250 mg/kg), Ce6 (5 mg/kg), and CFN-gel (5 mg Ce6 equivalent/kg). (**a**–**d**) Fluorescence images of the tumors and organs extracted 24 h after injection of 5-ALA, Ce6, and CFN-gel. (T: tumor, H: heart, S: spleen, K: kidney, L: liver, Lu: lung). Fluorescence images of 5-ALA were obtained using 405 nm, and that of Ce6 and CFN-gel were obtained using 650 nm.

As shown in Figure 5, to confirm the possibility of image-guided surgery of the proposed imaging system and the previously developed CFN-gel, xenograft mice injected with colorectal cancer cells were fabricated, and in vivo experiments were performed. Over time, cancer cell accumulation was observed by injecting commercially available 5-ALA, Ce6, and CFN-gel. In this study, the proposed near-infrared fluorescence diagnostic–therapy system was used to observe the accumulation of PpIX (5-ALA), Ce6, and CFN-gel in cancer, and a filter and light source appropriate for the absorption and excitation wavelengths of PpIX (5-ALA), Ce6, and CFN-gel were used. Unlike the cancer cell experiment, in the case of the in vivo experiment, the fluorescence efficiency was lower than that of the cell experiment because the mouse skin tissue had to be irradiated with light. Over time, 5-ALA has been shown to be eliminated from the body rather than significantly accumulating at tumor sites. In Figure 5B, it can be seen that strong fluorescence is emitted throughout the body at 4, 6, and 9 h in mice injected with 5-ALA. This is not the fluorescence from PpIX accumulated in the cancer but the autofluorescence from PpIX present on the skin surface. Similar to the results of the cancer cell experiment, it was found that Ce6 accumulated better in the cancerous region of xenograft mice than 5-ALA did. However, almost no fluorescence was observed after 24 h. Alternatively, in the case of CFN-gel, it was confirmed through real-time fluorescence imaging that it accumulated well only in the tumor, and it was confirmed that the fluorescence image after 24 h also appeared well. Unlike in cancer cell experiments, the fluorescence intensity decreases in animal experiments because the light is absorbed, scattered, and reflected from the skin. Since it is difficult to confirm the accumulation of PpIX (5-ALA), Ce6, and CFN-gel inside the mouse, each mouse was sacrificed after 24 h, and the tumors and organs (heart, spleen, kidney, liver, and lung) were removed to confirm fluorescence. In 5-ALA, no fluorescence signal was observed in tumors and organs, indicating that most PpIX (5-ALA), Ce6, and CFN-gel were released from the body after 24 h. However, in the case of Ce6, although the fluorescence intensity was low, a fluorescence signal was observed in the tumor. In the case of CFN-gel, not only did it have high fluorescence intensity in the tumor, but also a small amount of fluorescence signal was confirmed in the lung and liver. As such, CFN-gel was well-adsorbed to colorectal cancer and had a long blood circulation time, confirming the possibility of image-guided surgery through the proposed system.

### 3.4. Detection of Lymph Node Metastasis

**Figure 6:**
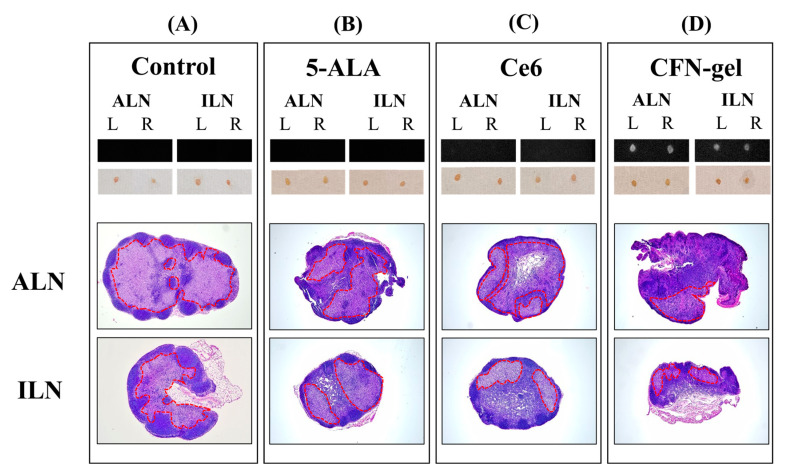
Detection of lymph node metastasis using CFN-gel and fabricated near-infrared fluorescence diagnostic–therapy system. (**A**–**D**) Lymph node ALN (axillary lymph node) and ILN (inguinal lymph node) were extracted after 24 h fluorescence imaging. The inside of the red dotted line indicates cancer. H&E staining conducted only fluorescence imaging side of the mouse model (control, 5-ALA, and Ce6: left side lymph node of the mouse model; CFN-gel: right side lymph node of the mouse). H&E staining images of lymph nodes were acquired at 4× lens magnification.

In the case of fluorescence using general indocyanine green, only real-time fluorescence images of the lymph nodes are displayed, but the metastasis of the lymph nodes cannot be confirmed [31,32]. In cancer treatment, the presence of lymph node metastasis is vital for cancer staging and affects the choice of proper therapy related to survival rates. Real-time detection of lymph node metastasis for resection is key to preventing cancer progression. Therefore, in this study, the possibility of diagnosis/treatment of colorectal cancer was confirmed using the proposed fluorescence imaging system and the previously developed CFN-gel. Lymph node metastasis was confirmed through fluorescence imaging and verified through H&E staining. Figure 6 shows the fluorescence and H&E staining images of lymph nodes extracted from mice 24 h after injecting 5-ALA, Ce6, and CFN-gel into the xenograft model. Clear fluorescence images were confirmed in the lymph nodes of the mouse model using CFN-gel. Despite the presence of cancer cells in the entire lymph node through H&E staining, the fluorescence of PpIX (5-ALA) and Ce6 was not observed, but CFN-gel made it possible to confirm lymph node metastasis in real time through clear fluorescence images. Therefore, it was confirmed that the combination of CFN-gel and the proposed near-infrared fluorescence diagnostic–therapy system could simultaneously perform colorectal cancer diagnosis, lymph node metastasis diagnosis, and treatment.

### 3.5. In Vivo PDT

**Figure 7:**
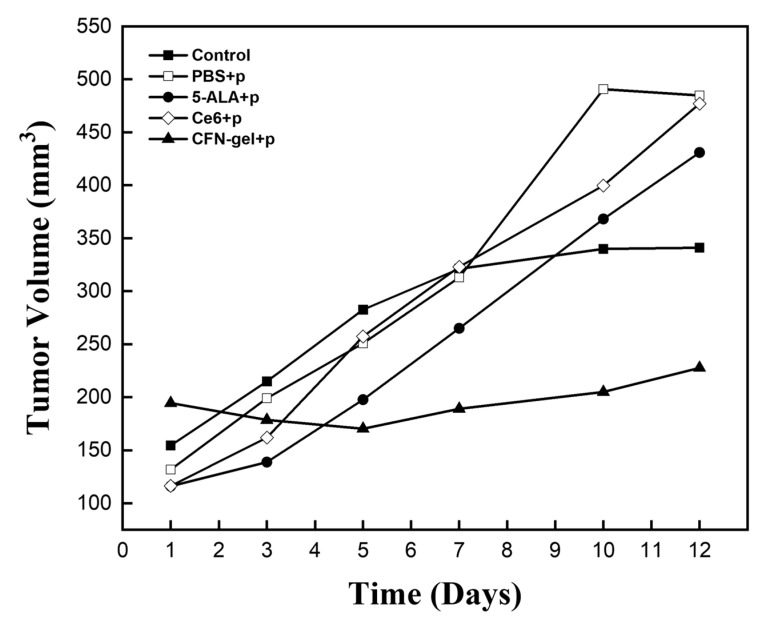
Changes in tumor volume in response to PDT. Control group, PBS + PDT, 5-ALA + PDT, Ce6 + PDT, and CFN-gel + PDT. PBS (100 μL), 5-ALA (250 mg/kg), Ce6 (5 mg/kg), and CFN-gel (5 mg Ce6 equivalent/kg) were intravenously injected on day 1, and PDT was conducted on day 2 (wavelength, 660 nm; fluence, 18 J/cm^2^).

Finally, the system was switched to a light source for treatment, and PDT was performed on the tumor area; the results are shown in Figure 7. PBS, 5-ALA, Ce6, and CFN-gel were intravenously injected into the xenograft mice, and PDT was performed 24 h later. The light used for PDT had a wavelength of 660 nm and an irradiation dose of 18 J/cm^2^ (irradiation for 6 min with an optical power of 50 mW/cm^2^). PDT was performed only once after 24 h, and the tumor volumes of the five groups subjected to PDT were observed for 12 days and displayed in a graph. After 24 h, it was confirmed that there was no or a weak fluorescence signal in the cancer cells treated with 5-ALA and Ce6 through the cancer cell images in Figure 2 and the in vivo images in Figure 5B,C. Similar to the results, in the PDT experiment performed after 24 h, 5-ALA and Ce6 combined xenograft mice did not show significant results and continued to grow in tumor size. However, in the case of CFN-gel, the size of the cancer decreased until the 5th day after PDT was performed; after the 5th day, the size of the cancer gradually increased, but the increase was significantly reduced compared with the other comparison groups. Based on these results, the proposed switchable near-infrared fluorescence diagnostic–therapy system and previously developed CFN-gel confirmed the possibility of image-guided surgery, lymph node metastasis diagnosis, and cancer treatment.

## 4. Conclusions and Discussion

In this study, we report the possibility of colorectal cancer image-guided surgery, lymph node metastasis of colorectal cancer cells, and suppression of tumor growth through PDT using a near-infrared fluorescence diagnostic–therapy system and the previously developed CFN-gel. First, three laser diodes of 405 nm, 650 nm, and 660 nm, which are the absorption and treatment wavelengths of PpIX (5-ALA), Ce6, and CFN-gel used in this study, respectively, were used to confirm the diagnosis and metastasis of colorectal cancer through fluorescence imaging. In addition, a near-infrared fluorescence diagnostic–therapy system was implemented using an optical switch such that diagnosis/treatment could be performed in the same optical path. In vitro experiments using HCT 116 colorectal cancer cells were performed using CFN-gel, and conventional 5-ALA and Ce6 were used for comparison. The fluorescence intensity over time of 5-ALA-, Ce6-, and CFN-gel-treated cells was obtained through images obtained with a confocal microscope and a self-made two-photon microscope. As a result, even after 24 h, CFN-gel had a higher fluorescence intensity than PpIX (5-ALA) and Ce6. In addition, in vitro cell studies were conducted to confirm the cancer cell treatment ability of PpIX (5-ALA), Ce6, and CFN-gel and the PDT effect in the cellular state. After concentrating HCT116 cells with 5-ALA, Ce6, and CFN-gel for 6 h, cell viability was confirmed in the non-irradiated and light-irradiated groups. In an in vitro study, no change was seen in the group not irradiated with light, and in the light-irradiated group, CFN-gel confirmed a significant decrease in cancer cell viability of 2 μM Ce6 equivalent or more. To confirm the proposed imaging system and CFN-gel’s potential for image-guided surgery, a xenograft mouse model injected with human colorectal cancer cells was prepared, and fluorescence images were successfully obtained. Unlike commercially available 5-ALA and Ce6, CFN-gel fluorescence was well-detected at the tumor site even after 24 h. After 24 h, the xenograft mice were sacrificed, and metastasis to the lymph nodes was confirmed. In the case of 5-ALA and Ce6, although cancer metastasis was confirmed through H&E staining, fluorescence images were not acquired. However, in the case of CFN-gel, fluorescence was also confirmed in the lymph nodes, demonstrating the possibility of real-time diagnosis of lymph node metastasis. Simultaneously with diagnosis, PDT was performed on xenograft mice by switching to a therapeutic light source. PDT was performed 24 h after the injection of the contrast agent. As a result of the observation for 12 days, only CFN-gel inhibited cancer cell proliferation.

As a result of PDT, it was confirmed that CFN-gel had a greater tumor suppression effect than other commercially available PpIX (5-ALA) and Ce6, but complete treatment was not confirmed. Treatment effects may vary depending on the type of cancer cells, and there is a possibility that the light source for treatment may not be properly delivered inside the tumor. In the future, it will be necessary to apply more related research to various cancer cells and study the therapeutic effect according to the optical power intensity.

Through this study, we confirmed the possibility of image-guided surgery and PDT in combination with the proposed system and CFN-gel. For this reason, the results of this study can become the core of precise image-guided surgery in the future and also have the potential to grow into a key technology that can check the metastasis of the operculum lymph node in real time.

## Data Availability

The data presented in this study are available upon request from the corresponding author.
